# Umbilical Cord-Derived Mesenchymal Stem Cells Relieve Hindlimb Ischemia through Enhancing Angiogenesis in Tree Shrews

**DOI:** 10.1155/2016/9742034

**Published:** 2016-08-29

**Authors:** Cunping Yin, Yuan Liang, Jian Zhang, Guangping Ruan, Zian Li, Rongqing Pang, Xinghua Pan

**Affiliations:** ^1^Department of Vascular Surgery, Kunming General Hospital, Chengdu Military Command, Kunming 650032, China; ^2^Stem Cell Engineering Laboratory of Yunnan Province, Kunming General Hospital, Chengdu Military Command, Kunming 650032, China; ^3^Department of Geriatrics, Kunming General Hospital, Chengdu Military Command, Kunming 650032, China; ^4^Department of Medical Imaging, Kunming General Hospital, Chengdu Military Command, Kunming 650032, China

## Abstract

Hindlimb ischemia is still a clinical problem with high morbidity and mortality. Patients suffer from consequent rest pain, ulcers, cool limbs, and even amputation. Angiogenesis is a promising target for the treatment of ischemic limbs, providing extra blood for the ischemic region. In the present study, we investigated the role of umbilical cord-derived mesenchymal stem cells (UC-MSCs) in regulating angiogenesis and relieving hindlimb ischemia. UC-MSCs were isolated from the umbilical cord of tree shrews. Angiography results showed that UC-MSCs injection significantly promoted angiogenesis in tree shrews. Moreover, the ankle brachial index, transcutaneous oxygen pressure, blood perfusion, and capillary/muscle fiber ratio were all markedly increased by the application of UC-MSCs. In addition, the conditioned culture of human umbilical vein endothelial cells using medium collected from UC-MSCs showed higher expression of angiogenic markers and improved migration ability. In short, the isolated UC-MSCs notably contributed to restoring blood supply and alleviating the symptoms of limb ischemia through enhancing angiogenesis.

## 1. Introduction

Limb ischemia caused by peripheral arterial disease is accompanied by a high morbidity and mortality rate of about 25% and an amputation rate of 30% [1]. Limb ischemia is characterized by rest pain, ulcers, cool limbs, gangrene, and absent distal peripheral pulses [2]. Current treatment options for limb ischemia rely on surgical revascularization and restoring blood supply to the limb [3], but they have a poor outcome due to the limited coverage to the distal limb and comorbidities including oxidative damage. Angiogenesis that aims to improve blood perfusion will be helpful for the treatment of limb ischemia with the hope of reducing the need for limb amputation.

In fact, the application of angiogenesis in the treatment of limb ischemia has emerged as a new therapeutic method recently. Angiogenesis and revascularization will provide increased blood supply and consequently oxygen and nutrients to the ischemic region, thus relieving the symptoms of hindlimb ischemia [4, 5]. For example, proangiogenic cytokines, such as vascular endothelial growth factor (VEGF) and basic fibroblast growth factor (bFGF), play a significant role in promoting angiogenesis accompanied by enhanced collateral circulation in patients with hindlimb ischemia [6]. It has also been reported that the transplantation of mesenchymal stem cells (MSCs) derived from bone marrow or placenta significantly facilitated angiogenesis in animals with hindlimb ischemia [3, 7]. Moreover, endothelial progenitor cells (EPCs) were reportedly able to refresh dysfunctional mature endothelial cells incorporated into blood cells [8]. Actually, the proangiogenic cytokines released from MSCs could promote angiogenic differentiation, leading to the stimulation of resident EPCs to migrate, proliferate, and differentiate into incorporated endothelial cells* in vivo* [9].* In vitro*, MSCs could also secrete VEGF and other proangiogenic factors that can promote the migration, proliferation, and tube formation of endothelial cells [10]. In conclusion, among various types of stem cells, bone marrow-derived MSCs (BM-MSCs) and EPCs are the two most widely used sources of seed cells for ischemia treatment [11]. However, due to the fact that BM-MSCs and EPCs originate from adult tissues, the limited proliferative and functional ability of these two cell lines impedes their therapeutic application [12]. These limitations have necessitated investigation of other sources for stem cell therapy.

Umbilical cord-derived MSCs (UC-MSCs) are believed to be extremely helpful for tissue remodeling and regeneration because their proliferative ability has not been dampened by age and disease, and they are free from acquired pathogens [13, 14]. In addition, UC-MSCs might reduce the risk of immunorejection [15], thus offering huge advantages for therapeutic application over other types of stem cells [16], mainly due to the fact that the umbilical cord tissues are cryopreserved immediately after birth for possible future autologous use [17]. The therapeutic potential of UC-MSCs for the treatment of ischemic diseases has been demonstrated in clinical trials [18, 19]. Moreover, Cheng et al. reported that the administration of human UC-MSCs resulted in significant therapeutic effects in the ischemic brain in mice [20]. Another study reported that transplantation of gene-infected UC-MSCs significantly relieved hindlimb ischemia in rats [21].

In short, stem cell-based approaches have drawn more attention because stem cells could be directionally induced to differentiate and, theoretically, form new blood vessels [11, 22]. In our previous study, we used BM-MSCs to investigate their role in a rat model of hindlimb ischemia. In the present study, we hypothesized that UC-MSCs could be used to treat limb ischemia. UC-MSCs were employed to explore their role in regulating hindlimb ischemia and angiogenesis in a tree shrew model, which aimed to provide further experimental basis for the therapeutic application of MSCs in limb ischemia.

## 2. Materials and Methods

### 2.1. Animal Feeding and Hindlimb Ischemia Model

Tree shrews were purchased from the Kunming General Hospital and housed under pathogen-free conditions with constant temperature (37°C), regular feeding, and free access to sterilized drinking water. All animal experimental procedures conformed to the guidelines issued by the Kunming General Hospital, Chengdu Military Command for Laboratory Animals. The present study was performed with approval from the Animal Ethics Committee of the Kunming General Hospital, Chengdu Military Command (certification number 0119). All animals were subjected to anesthesia with intraperitoneal injection of sodium pentobarbital prior to experiments (40 mg/kg) to relieve the pain. For constructing the hindlimb ischemia model, the left femoral vessels were excised without damaging the femoral nerve. Briefly, a longitudinal incision was made along the left medial thigh. The proximal portion of the femoral artery and the distal portion of the saphenous artery were ligated with silk ligatures. The remaining muscular branches between these two sites as well as veins were all dissected and excised. The corresponding right hindlimb was kept intact and used as the nonischemic limb. Measurements were performed on days 0, 7, and 14 after surgery to evaluate the model construction. All animals survived without infections or necrosis in the ischemic hindlimbs.

### 2.2. Isolation of UC-MSCs

MSCs were isolated from the umbilical cord of adult pregnant tree shrews (*n* = 6, 120–140 g). Briefly, the full-term pregnant tree shrews (8 weeks) were sacrificed via breaking the neck and sterilized with 75% ethanol. Abdominal cavity was then opened, and uterus was cut open. Neonatal shrews and placenta were therefore exposed. Subsequently, umbilical cord was cut off and immediately rinsed in DMEM/F12 medium (Hyclone, Logan, UT, USA) supplemented with 100 units/mL penicillin-streptomycin (Hyclone). Next, the needless tissues, such as umbilical artery and umbilical vein, were separated from umbilical cord and discarded. The acquired umbilical cord was then cut into pieces (approximately 1 × 1 × 1 mm^3^) and centrifuged at 1500 ×g for 5 min, followed by being resuspended in DMEM/F12 medium containing 20% fetal bovine serum (FBS, Hyclone) and centrifuged at 1500 ×g for 5 min again. Upon that, the tissue explants were resuspended and seeded uniformly into culture flasks containing DMEM/F12 medium supplemented with 20% FBS, 100 units/mL penicillin-streptomycin, 2 mM L-glutamine (Hyclone), and 1% nonessential amino acids (Hyclone) in a humidified atmosphere of 37°C with 5% CO_2_. The medium was completely replaced every 3 days, and the nonadherent cells were discarded. MSCs were characterized by their highly proliferative ability with spindle-shaped and well-spread morphology. Cells were subcultured at a 1 : 3 dilution into culture dishes using 0.25% Trypsin (Hyclone) when reaching a confluence of over 80%. Cells were observed and photographed using an Olympus microscope (DSX110, Osaka, Japan).

### 2.3. Flow Cytometry and Cell Characterization

MSCs at passage 3 were digested and resuspended in 100 *μ*L of PBS with a density of 1 × 10^5^ cells. The cells were labeled with FITC-conjugated antibodies (BD Biosciences, San Diego, USA), including CD13, CD34, CD45, CD73, CD90, and CD105. FITC-conjugated isotype-matching IgS was used to determine nonspecific staining. The cells were examined using a FACSCalibur cytometer (BD Biosciences), and the data were analyzed using Cytosoft version 5.2 (Guava Technologies, Hayward, CA, USA).

### 2.4. MTT Assay

MSCs at passage 3 were digested and seeded into 96-well plates with a density of 1.5 × 10^4^ cells/well. MTT solution was added to indicated wells and incubated for 48 h, according to the manufacturer's instructions for the MTT Cell Proliferation and Cytotoxicity Assay Kit (Beyotime, Shanghai, China). The mixtures were replaced with dimethylsulfoxide (DMSO) to dissolve the formazan. The optical density (OD) value at 490 nm was recorded.

### 2.5. UC-MSC Injection and Animal Grouping

Cultured UC-MSCs at passage 3 were detached from culture dishes using Trypsin (Hyclone), followed by injection into the caudal vein of tree shrews on the first day after surgery. Tree shrews were randomly divided into five groups: (1) tree shrews fed without any treatments (control group, *n* = 10), (2) tree shrews with hindlimb ischemia (sham group, *n* = 10), (3) hindlimb ischemia with PBS injection (negative control, NC group, *n* = 10), (4) hindlimb ischemia with injection of 2 × 10^6^ MSCs (2 × 10^6^ group, *n* = 10), and (5) hindlimb ischemia with injection of 6 × 10^6^ MSCs (6 × 10^6^ group, *n* = 10). Measurements were performed on days 7, 14, 21, and 28 after surgery.

### 2.6. Angiography (Computed Tomography Angiography, CTA)

The tree shrews were subjected to angiography (arterial CTA) 21 days after surgery to monitor angiogenesis* in vivo*. Briefly, animals were anesthetized and fixed to the sample table. The abdominal cavity was opened, and a catheter was inserted downward from the abdominal aorta for the injection of the contrast medium. The animals were transferred onto a SOMATOM Spirit Scanner (Siemens Healthcare, Erlangen, Germany), and the X-ray was adjusted to the target area. The settings are as follows: Smart model (tracking), 27 seconds (scanning), and 0.1 mL/second (flow rate).

### 2.7. Ankle Brachial Index (ABI)

ABI was assessed using a noninvasive sphygmomanometer (BP98A, Softron Biotechnology, Beijing, China). Briefly, the systolic blood pressure of the posterior tibial artery and brachial artery was measured, respectively. The ratio of average posterior tibial artery pressure versus brachial artery pressure was calculated and regarded as the ABI.

### 2.8. Transcutaneous Oxygen Pressure (TcPO_2_)

The TcPO_2_ was measured on the dorsal parts in the crus and thigh of hindlimbs using a TcPO_2_ analyzer (PF5000, Perimed, Stockholm, Sweden) as previously described [12]. The value of mmHg was set as the *y*-axis.

### 2.9. Blood Perfusion

A laser Doppler perfusion image (LDPI) analyzer (OMEGA ZONE OZ-1, Omega Wave, Inc., Tokyo, Japan) was employed to measure the blood flow as previously described [12]. The perfusion value was measured using LDISOFT software. The ratio of the average perfusion value in ischemic versus nonischemic limbs was calculated and regarded as the LDPI index.

### 2.10. Capillary/Muscle Fiber Ratio

Capillary density was measured as previously described [12]. Briefly, tissue specimens were collected from adductor muscles, frozen in liquid nitrogen and cut into 5 *μ*m slices, and then stained for alkaline phosphatase to detect capillary endothelial cells. The ratio of the average area of capillary endothelial cells versus muscle fiber was calculated.

### 2.11. Conditioned Culture of Human Umbilical Vein Epithelial Cells (HUVECs)

HUVECs were purchased from the China Center of Type Culture Collection (CCTCC, Wuhan, China) and cultured in RPMI-1640 medium (Hyclone) supplemented with 10% FBS (Hyclone) in a humidified atmosphere of 37°C with 5% CO_2_. For the conditioned culture, the medium was replaced with the conditioned medium (CM), which was collected from UC-MSCs cultured in low glucose DMEM and filtered using a 0.45 *μ*m filter. HUVECs were cultured in CM for 72 h (CM group).

### 2.12. Cell Migration

The migration ability of UC-MSC-conditioned-cultured HUVECs was assessed using the wound repair assay. Briefly, cells were pretreated with Mitomycin C (10 *μ*g/mL, Sigma-Aldrich) for 2 h prior to wound repair assay, aiming to inhibit its proliferation for an equal assessment of cell migration. Wounds were created by dragging a sterile pipette tip across the monolayer, creating a 350 mm cell-free path. Twenty-four hours later, the decreased wound area/original wound area ratio was calculated.

### 2.13. Real-Time Quantitative PCR

The total RNA from HUVECs was extracted using TRIzol® Reagent (Invitrogen, Carlsbad, CA, USA) and cDNA was synthesized through RNA reverse transcription using PrimeScript*™* II 1st-Strand cDNA Synthesis Kit (TaKaRa Biotechnology, Dalian, China) following the manufacturer's instructions. Quantitative PCR was performed using SYBR® Fast qPCR Mix (TaKaRa Biotechnology) according to the manufacturer's instructions. The primer sequences and fragment sizes used are listed in Table 1. GAPDH served as the internal reference gene. The fold expression relative to GAPDH was calculated using 2^−ΔΔCt^ method.

### 2.14. Western Blot Analysis

For protein extraction of HUVECs, the medium was discarded and RIPA buffer (Sigma-Aldrich) was added, followed by rotation on ice for 15 min. The mixtures were scraped from culture dishes into a centrifuge tube and subjected to repetitive shaking for dissolving. The proteins were quantified using the Bradford method. A total of 30 *μ*g of protein was subjected to 10% SDS-PAGE and then electrotransferred onto a PVDF membrane (Merck Millipore, Billerica, MA, USA). The membrane was incubated with primary antibodies and horseradish peroxidase- (HRP-) conjugated secondary antibody (Invitrogen), successively. Bands were detected using an enhanced chemiluminescence kit (Thermo Fisher Scientific, Waltham, MA, USA). Detection of GAPDH was used as the internal reference.

### 2.15. Statistical Analysis

Data were collected from at least three independent experiments and presented as mean ± standard deviation (SD). Statistics were performed using GraphPad Prism 5 software. Student's* t*-test was employed to compare the data between two groups; for multiple group comparison, analysis of variance (ANOVA) test was performed. *p* < 0.05 and *p* < 0.01 were considered to be statistically significant.

## 3. Results

### 3.1. Characterization of UC-MSCs

**Figure 1:**
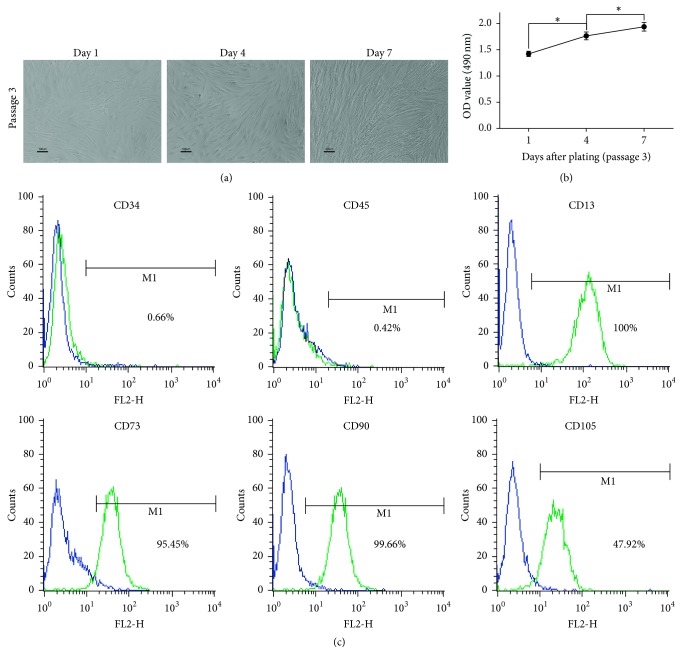
Characterization of UC-MSCs. (a) Morphological changes and characterization of UC-MSCs. (b) The viability of UC-MSCs after plating from day 1 to day 7 measured by MTT assay. (c) The expression of cell surface markers measured by flow cytometry. UC-MSCs positively expressed CD13, CD73, CD90, and CD105 but negatively expressed CD34 and CD45. *∗* represents *p* < 0.05.

The morphology of isolated UC-MSCs is shown in Figure 1(a). UC-MSCs demonstrated a short spindle-shaped morphology on the first day after plating. Long spindle-shaped UC-MSCs were formed 4 days later and numerous spindle-shaped UC-MSCs appeared 7 days after plating. Moreover, UC-MSCs had an increasing viability as shown in Figure 1(b). The cell surface markers were measured by flow cytometry. UC-MSCs were more than 80% positive for CD13, CD73, and CD90 and about 50% positive for CD105 but less than 1% positive for CD34 and CD45 (Figure 1(c)), indicating that the isolated UC-MSCs had a typical MSC immune-phenotype.

### 3.2. Construction of the Hindlimb Ischemia Model

**Figure 2:**
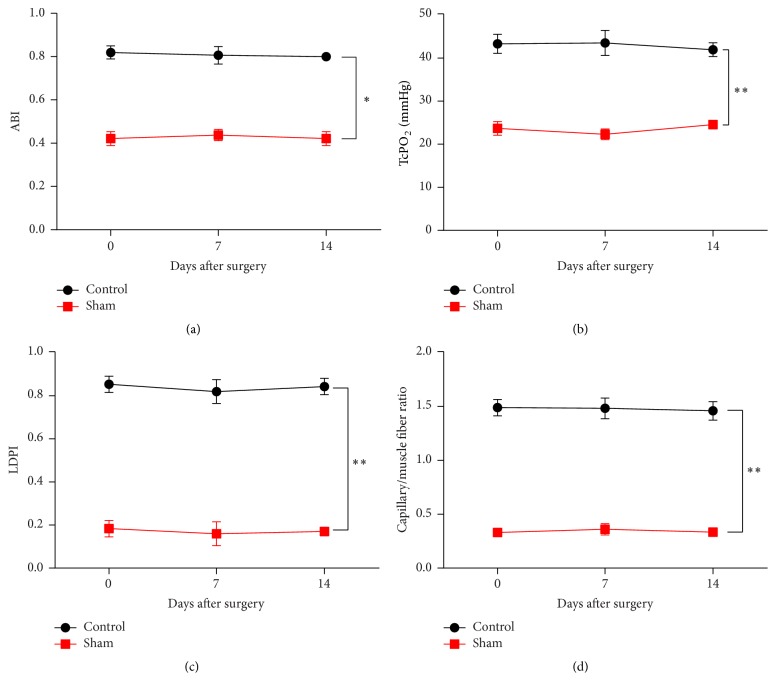
Evaluation of hindlimb ischemia model. (a) ABI, (b) TcPO_2_, (c) LDPI, and (d) capillary/muscle fiber ratio were all significantly decreased in the sham group compared with those in the control group. Data are presented as means ± SD from three independent experiments. *∗* represents *p* < 0.05 and *∗∗* represents *p* < 0.01.

Here we aimed to determine whether the ischemic hindlimb model was constructed effectively and successfully. ABI (Figure 2(a)), TcPO_2_ (Figure 2(b)), LDPI (Figure 2(c)), and capillary/muscle fiber ratio (Figure 2(d)) in the sham group were all remarkably decreased on days 7 and 14 after surgery compared with the data in the control group, confirming the effectiveness of the hindlimb ischemia model.

### 3.3. UC-MSC Therapy Relieves Hindlimb Ischemia

**Figure 3:**
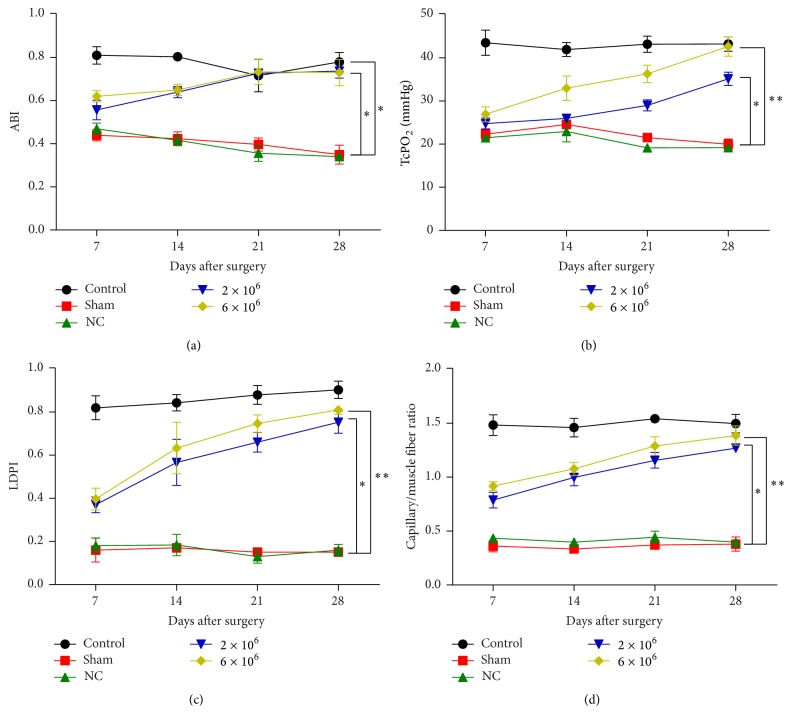
UC-MSCs relieved hindlimb ischemia. (a) ABI, (b) TcPO_2_, (c) LDPI, and (d) capillary/muscle fiber ratio were increased after injection of UC-MSCs. Data are presented as means ± SD from three independent experiments. *∗* represents *p* < 0.05 and *∗∗* represents *p* < 0.01 of sham group versus 2 × 10^6^ or 6 × 10^6^ groups.

In this part, UC-MSCs were injected into tree shrews to evaluate the effect on limb ischemia. ABI was significantly and continuously increased after UC-MSC therapy compared with the sham group (Figure 3(a)). Similarly, TcPO_2_ was also increased upon UC-MSC injection (Figure 3(b)), possibly due to the restored blood supply as shown in Figure 3(c). The capillary/muscle fiber ratio was improved during the 28-day period after UC-MSC injection (Figure 3(d)). Besides this, injection of 6 × 10^6^ UC-MSCs was more effective compared to 2 × 10^6^ UC-MSCs, and all these four indexes were restored to a comparable level to control group after UC-MSCs injection. These results indicate that UC-MSCs contribute to attenuating hindlimb ischemia.

### 3.4. UC-MSCs Therapy Promotes Angiogenesis

**Figure 4:**
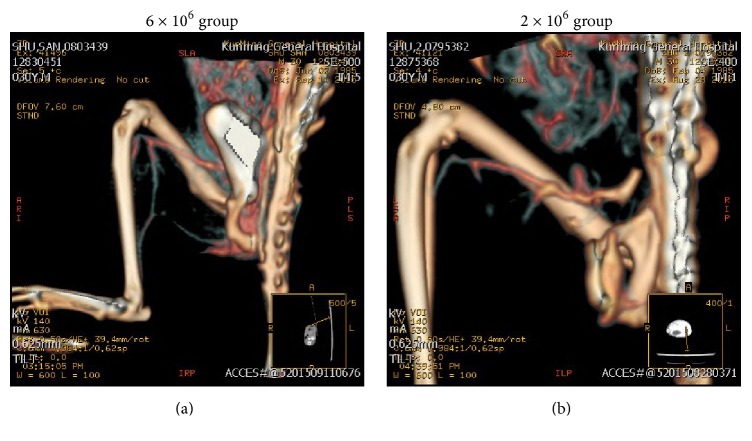
Angiography (arterial CTA). Angiography was performed 21 days postoperatively. (a) The injection of 6 × 10^6^ UC-MSCs significantly promoted angiogenesis. (b) Injection of 2 × 10^6^ UC-MSCs showed less effectiveness.

Arterial CTA was performed postoperatively to visualize angiogenesis* in vivo*. As shown in Figure 4(a), in the 6 × 10^6^ group, a renascent and intact blood vessel had been formed in the tree shrew hindlimb. However, the injection of 2 × 10^6^ UC-MSCs demonstrated less and segmental blood vessel (Figure 4(b)). These results confirm the role of UC-MSCs in promoting angiogenesis.

### 3.5. Conditioned Culture of HUVECs Promotes Their Migration and Angiogenic Differentiation

**Figure 5:**
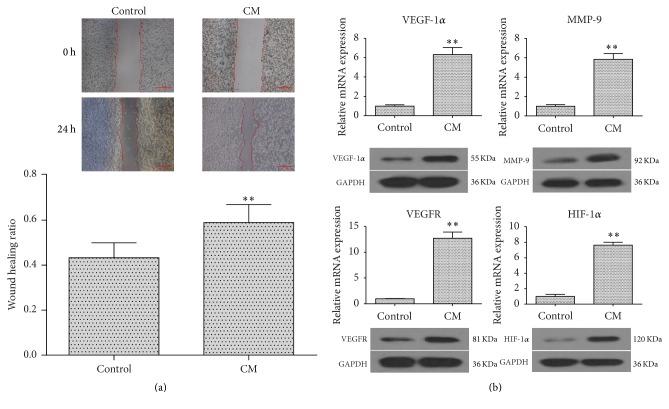
Conditioned culture of HUVECs promoted their migration and angiogenic differentiation. HUVECs were cultured in CM for 72 h. (a) The significantly reduced wound area by CM. (b) The markedly upregulated expression of VEGF-1*α*, MMP-9, VEGFR, and HIF-1*α* by CM. Data are presented as means ± SD from three independent experiments. *∗∗* represents *p* < 0.01; scale bars: 500 *μ*m.

The CM collected from UC-MSC culture dishes was used to subculture HUVECs. Migration ability and angiogenic marker expression were investigated. The results indicated that CM significantly increased the capacity for migration in HUVECs compared with the control group (Figure 5(a)). In addition, the CM-cultured HUVECs showed markedly higher expression of angiogenic markers, including vascular endothelial growth factor 1*α* (VEGF-1*α*), matrix metalloproteinase 9 (MMP-9), vascular endothelial growth factor receptor (VEGFR), and hypoxia-inducible factor 1*α* (HIF-1*α*), compared to the control group (Figure 5(b)). These results further confirm the proangiogenic effects of UC-MSCs.

## 4. Discussion

In the present study, with the help of a hindlimb ischemia model in tree shrews, we investigated the role of UC-MSCs in regulating angiogenesis. The results showed that therapy with UC-MSCs could significantly relieve hindlimb ischemia accompanied by enhanced angiogenesis* in vivo* and* in vitro*, inspiring us to develop new drugs for limb ischemia based on these discoveries.

MSCs have mesodermal abilities due to their mesodermal derivation from bone marrow [23], placenta [24], umbilical cord blood [25], and adipose tissues [26], which means that MSCs tend to differentiate into originally mesoderm-derived cells, such as osteoblasts, chondrocytes, and adipocytes [27]. These characteristics enable MSCs to be an ideal candidate for mesodermal tissue repair. Vascular endothelial cells also belong to the mesodermal derivatives, which enlightened us that MSCs should be used to promote angiogenesis and ultimately treat ischemia. Actually, the application of MSCs has been already reported to be able to accelerate angiogenesis [28]. UC-MSCs are more promising candidates among various types of MSCs for autologous tissue repair and vessel regeneration, because they are highly proliferative, rarely affected by pathogens and viruses, and poorly immunorejective. In our study, the isolated UC-MSCs had a typical immunophenotype, including positive expression of CD13, CD73, CD90, and CD105 and negative expression of CD34 and CD45. As reported, CD13 and CD105 are MSC markers; CD73 is an endothelial cell and stem cell marker; CD90 is a thymocyte differentiation antigen [7, 29]. Thus, the positive expression of CD13, CD73, CD90, and CD105 confirmed the typical attributes of MSCs in the isolated and primarily cultured UC-MSCs.

For the animal model in this study, we used tree shrews instead of the previously used rat model, mainly due to the fact that the tree shrew (*Tupaia belangeri*) is similar to humans genetically and anatomically [30]. Moreover, its metabolism is more similar to humans compared to mice or rats and so forth [31]. Hence, the study on tree shrews will be more meaningful to humans. In this study, ABI, TcPO_2_, LDPI, and capillary/muscle fiber ratio were all measured on tree shrews. ABI is an indicator of intermittent claudication (0.35 < ABI < 0.9) and rest pain (ABI < 0.4), which are two major symptoms of limb ischemia. TcPO_2_ and LDPI are two indicators of blood perfusion that aim to determine the blood flow and oxygen supply. Capillary/muscle fiber ratio is a reflection of vessel formation. The results showed that, in UC-MSC-treated tree shrews (6 × 10^6^ group), ABI was recovered 28 days postoperatively (ABI = 0.73 ± 0.06); TcPO_2_ and LDPI were also restored to a normal level 28 days later (TcPO_2_ = 42.65 ± 2.25; LDPI = 0.81 ± 0.02); capillary/muscle fiber ratio was significantly increased compared with the sham group (capillary/muscle fiber ratio = 1.39 ± 0.09). In total, the above results confirmed the effects of UC-MSCs on mitigating hindlimb ischemia. Collectively, the results of the current study support our hypothesis that UC-MSCs could be used to relieve limb ischemia, accompanied by enhanced angiogenesis.

## 5. Conclusion

This study successfully isolated UC-MSCs from tree shrews that expressed specific cell surface markers including CD13, CD73, CD90, and CD105, and UC-MSCs could relieve hindlimb ischemia effectively and efficiently. Moreover, UC-MSCs were proved to promote angiogenesis* in vivo* and* in vitro*, as demonstrated by angiography and high expression of VEGF-1*α*, MMP-9, VEGFR, and HIF-1*α*. The novel results might provide a promising therapeutic method for relieving and even treating limb ischemia. This study also suggests to us that we will repeat these experiments in clinical patients.

## Figures and Tables

**Table 1:** The primers' sequences for real-time quantitative PCR.

Gene	Primers	Product (bp)
VEGF-1*α*	CTGTCTAATGCCCTGGAGCC	124
	ACGCGAGTCTGTGTTTTTGC	

MMP-9	TTTGAGTCCGGTGGACGATG	153
	TTGTCGGCGATAAGGAAGGG	

VEGFR	CGGTCAACAAAGTCGGGAGA	123
	CAGTGCACCACAAAGACACG	

HIF-1*α*	ACTTGGCAACCTTGGATTGG	190
	ATCTCCGTCCCTCAACCTCT	

GAPDH	AATGGGCAGCCGTTAGGAAA	168
	GCGCCCAATACGACCAAATC	
